# Ethyl 3-ferrocenyl-1-(pyridin-2-ylmeth­yl)-1*H*-pyrazole-5-carboxyl­ate

**DOI:** 10.1107/S160053681105238X

**Published:** 2011-12-10

**Authors:** Ilia A. Guzei, Lara C. Spencer, Apollinaire Munyaneza, James Darkwa

**Affiliations:** a Department of Chemistry, University of Wisconsin-Madison, 1101 University Ave, Madison, WI 53706, USA; b Department of Chemistry, University of Johannesburg, Auckland Park Kingsway Campus, Johannesburg 2006, South Africa

## Abstract

The title compound, [Fe(C_5_H_5_)(C_17_H_16_N_3_O_2_)], crystallizes with an essentially eclipsed conformation of the cyclo­penta­dienyl (Cp) rings. The unsubstituted ring is disordered over two positions with the major component being present 90 (1)% of the time. The substituted Cp ring, the pyrazole ring and three atoms of the eth­oxy­carbonyl group form a conjugated π-system. These 13 atoms are coplanar within 0.09 Å.

## Related literature

For the preparation of (pyrazol-1-ylmeth­yl)pyridine compounds, see: House *et al.* (1986 ▶). For modification of the chemistry of metal (pyrazol-1-ylmeth­yl)pyridine compounds due to the substituents on the pyrazolyl ring, see: Ojwach *et al.* (2007 ▶, 2009 ▶). Typical structural parameters were confirmed by a *Mogul* geometry check, see: Bruno *et al.* (2002 ▶). Fe(II)–centroid distances for related compounds were found in the Cambridge Structural Database, see: Allen (2002 ▶). For discussion of the twinning of a nickel complex utilizing (3-ferrocenyl-5-ethyl­carboxyl­ate-pyrazolyl-1-yl-meth­yl)pyridine as a ligand, see: Guzei *et al.* (2012 ▶).
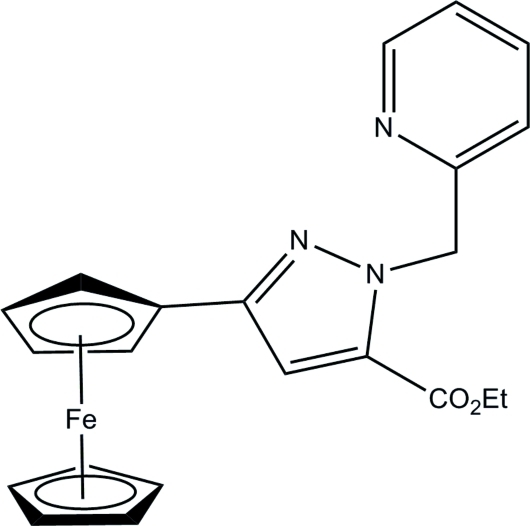


## Experimental

### 

#### Crystal data

[Fe(C_5_H_5_)(C_17_H_16_N_3_O_2_)]
*M* *_r_* = 415.27
Triclinic, 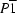

*a* = 10.3913 (2) Å
*b* = 10.6343 (2) Å
*c* = 10.7371 (2) Å
α = 86.6909 (8)°
β = 65.5907 (7)°
γ = 63.8778 (7)°
*V* = 958.82 (3) Å^3^
*Z* = 2
Mo *K*α radiation
μ = 0.81 mm^−1^
*T* = 296 K
0.35 × 0.28 × 0.21 mm

#### Data collection

Bruker SMART APEXII area-detector diffractometer
Absorption correction: analytical (*SADABS*; Bruker, 2011) ▶ *T*_min_ = 0.765, *T*_max_ = 0.851
19702 measured reflections
3852 independent reflections
3672 reflections with *I* > 2σ(*I*)
*R*_int_ = 0.020

#### Refinement

*R*[*F* ^2^ > 2σ(*F* ^2^)] = 0.025
*wR*(*F* ^2^) = 0.068
*S* = 1.05
3852 reflections
261 parameters
H-atom parameters constrained
Δρ_max_ = 0.23 e Å^−3^
Δρ_min_ = −0.16 e Å^−3^

### 

Data collection: *APEX2* (Bruker, 2011 ▶); cell refinement: *SAINT-Plus* (Bruker, 2011 ▶); data reduction: *SAINT-Plus*; program(s) used to solve structure: *SHELXTL* (Sheldrick, 2008 ▶); program(s) used to refine structure: *SHELXTL*, OLEX2 (Dolomanov *et al.*, 2009 ▶), *FCF_filter* (Guzei, 2007 ▶) and *INSerter* (Guzei, 2007 ▶); molecular graphics: *SHELXTL* and *DIAMOND* (Brandenburg, 1999 ▶); software used to prepare material for publication: *SHELXTL*, *publCIF* (Westrip, 2010 ▶) and *modiCIFer* (Guzei, 2007 ▶).

## Supplementary Material

Crystal structure: contains datablock(s) global, I. DOI: 10.1107/S160053681105238X/im2331sup1.cif

Structure factors: contains datablock(s) I. DOI: 10.1107/S160053681105238X/im2331Isup2.hkl

Additional supplementary materials:  crystallographic information; 3D view; checkCIF report

## Figures and Tables

**Fig. 1.**
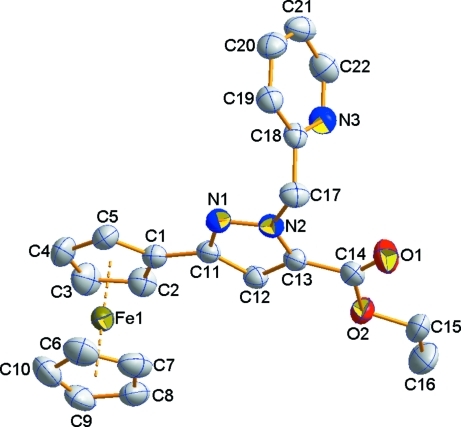
Molecular structure of (I) (Brandenburg, 1999). Thermal ellipsoids are shown at the 50% probability level. All hydrogen atoms were omitted for clarity.

## supplementary crystallographic information

### Comment

(Pyrazol-1-ylmethyl)pyridine compounds have been used as ligands in coordination chemistry since they were first prepared by House *et al.* in 1986. The chemistry of their metal compounds can be modified electronically and sterically by a careful selection of the substituents on the pyrazolyl ring (Ojwach *et al.*, 2007; Ojwach *et al.*; 2009). Herein we report the structure of the title compound (I).

The crystal structure of (I) was determined at room temperature because the crystals cracked upon flash- and slow-cooling to 100 K. The structural parameters of (I) are typical as confirmed by a *Mogul* geometry check (Bruno *et al.*, 2002). The Fe1—centroid(C1—C5), Fe1—Centroid(C6—C10), and Fe1—centroid(C6A—C10A) distances measure 1.643 (2), 1.651 (2), and 1.61 (2) Å, respectively, and agree well with Fe(II)—centroid distances reported for relevant complexes in the Cambridge Structural Database (August 2011, Allen (2002)).

The Cp ligands in the ferrocenyl unit are close to being eclipsed. The unsubstituted Cp ring is disordered over two positions with the major component being occupied 90 (1)% of the time. The two positions of the disordered Cp ring are on both sides of the ideally eclipsed position of the rings. The C1—centroid(C1—C5)—centroid(C6—C10)—C7 torsion angle involving the major Cp component is 11.5 (2)°; the corresponding C1—centroid(C1—C5)—centroid(C6a—C10*a*)—C7a to the minor component is -14.76 (6)°. The torsion angle between the substituted Cp ring and the attached pyrazolyl ligand is 9.91 (6)°; the torsion angle between the pyrazolyl ligand and the plane defined by atoms C14,O1,O2 is 3.66 (9)°; thus these three fragments form a conjugated system.

We also note that compound (I) was a ligand in a Ni(II) complex {Bromo[di-(3-ferrocenyl-5-ethylcarboxylate-pyrazolyl-1-ylmethyl)pyridine]nickel(II)} tetrabromoferrate(III) that showed a very interesting example of pseudo-merohedral twinning. The twinning was scrupulously discussed by Guzei *et al.* (2012).

### Experimental

To a benzene solution (20 ml) of 3-ferrocenyl-5-ethylcarboxylate-*1H*-pyrazole (0.11 g, 0.436 mmol) were added 2-picolylchloride hydrochloride (0.07 g, 0.436 mmol), 40% NaOH (12 ml) and 10 drops of 40% tertabutylammonium bromide (TBAB). The reaction mixture was refluxed for 18 h. The two phases were then separated and the organic phase dried over MgSO_4_. The solvent was removed in *vacuo* and the residue purified by preparative TLC (1: 2 petroleum ether/ethyl acetate). Yield: (0.030 g, 21%). Single crystals were obtained by slow evaporation of a solution of (I) in dichloromethane-toluene (3:1) solvent system at room temperature.

### Refinement

All H-atoms were placed in idealized locations and refined as riding with appropriate thermal displacement coefficients *U*_iso_(H) = 1.2 times *U*_eq_(bearing atom) for aromatic and methylene H atoms, and 1.5 times *U*_eq_(bearing atom) for the methyl H atoms.. The C—H distances were fixed at 0.93, 0.96, and 0.97 Å for aromatic, methyl, and methylene H atoms respectively. The C6a—C10*a* ring was refined with an idealized geometry. The thermal dsiplacement parameters for atoms C6a—C10*a* were contrained to be identical to those of atoms C6—C10, respectively.

### Crystal data

**Table:**

[Fe(C_5_H_5_)(C_17_H_16_N_3_O_2_)]	*Z* = 2
*M**_r_* = 415.27	*F*(000) = 432
Triclinic, *P*1	*D*_x_ = 1.438 Mg m^−^^3^
Hall symbol: -P 1	Mo *K*α radiation, λ = 0.71073 Å
*a* = 10.3913 (2) Å	Cell parameters from 9889 reflections
*b* = 10.6343 (2) Å	θ = 2.1–26.4°
*c* = 10.7371 (2) Å	µ = 0.81 mm^−^^1^
α = 86.6909 (8)°	*T* = 296 K
β = 65.5907 (7)°	Block, orange
γ = 63.8778 (7)°	0.35 × 0.28 × 0.21 mm
*V* = 958.82 (3) Å^3^	

### Data collection

**Table:**

Bruker SMART APEXII area-detector diffractometer	3672 reflections with *I* > 2σ(*I*)
mirror optics	*R*_int_ = 0.020
0.50° ω and 0.5° φ scans	θ_max_ = 26.4°, θ_min_ = 2.1°
Absorption correction: analytical (*SADABS*; Bruker, 211)	*h* = −12→12
*T*_min_ = 0.765, *T*_max_ = 0.851	*k* = −13→13
19702 measured reflections	*l* = −13→13
3852 independent reflections	

### Refinement

**Table:**

Refinement on *F*^2^	Primary atom site location: structure-invariant direct methods
Least-squares matrix: full	Secondary atom site location: difference Fourier map
*R*[*F*^2^ > 2σ(*F*^2^)] = 0.025	Hydrogen site location: inferred from neighbouring sites
*wR*(*F*^2^) = 0.068	H-atom parameters constrained
*S* = 1.05	*w* = 1/[σ^2^(*F*_o_^2^) + (0.0332*P*)^2^ + 0.246*P*] where *P* = (*F*_o_^2^ + 2*F*_c_^2^)/3
3852 reflections	(Δ/σ)_max_ = 0.001
261 parameters	Δρ_max_ = 0.23 e Å^−^^3^
0 restraints	Δρ_min_ = −0.16 e Å^−^^3^

### Special details

**Table:**

Experimental. 1H NMR (CDCl3): δ 1.43 (t, 3H, OCH2CH3), 4.07 (s, 5H, Fc), 4.27 (s, 2H, Fc), 4.41 (s, 2H, Fc), 4.46 (q, 2H, OCH2CH3), 5.76 (s, 2H, CH2py), 6.82 (d, 1H, py), 6.93 (s, 1H, pz), 7.22 (t, 1H, py), 7.63 (t, 1H, py), 8.62 (d, 1H, py).
Geometry. All e.s.d.'s (except the e.s.d. in the dihedral angle between two l.s. planes) are estimated using the full covariance matrix. The cell e.s.d.'s are taken into account individually in the estimation of e.s.d.'s in distances, angles and torsion angles; correlations between e.s.d.'s in cell parameters are only used when they are defined by crystal symmetry. An approximate (isotropic) treatment of cell e.s.d.'s is used for estimating e.s.d.'s involving l.s. planes.
Refinement. Refinement of *F*^2^ against ALL reflections. The weighted *R*-factor *wR* and goodness of fit *S* are based on *F*^2^, conventional *R*-factors *R* are based on *F*, with *F* set to zero for negative *F*^2^. The threshold expression of *F*^2^ > σ(*F*^2^) is used only for calculating *R*-factors(gt) *etc*. and is not relevant to the choice of reflections for refinement. *R*-factors based on *F*^2^ are statistically about twice as large as those based on *F*, and *R*- factors based on ALL data will be even larger.

### Fractional atomic coordinates and isotropic or equivalent isotropic displacement parameters (Å^2^)

**Table:**

	*x*	*y*	*z*	*U*_iso_*/*U*_eq_	Occ. (<1)
Fe1	0.28200 (2)	0.18372 (2)	0.366315 (18)	0.04291 (8)	
O1	0.68655 (14)	0.32795 (13)	−0.32948 (12)	0.0623 (3)	
O2	0.42373 (12)	0.45284 (12)	−0.20210 (11)	0.0523 (3)	
N1	0.65978 (13)	0.19596 (12)	0.05966 (12)	0.0420 (2)	
N2	0.69717 (12)	0.21986 (12)	−0.07203 (12)	0.0401 (2)	
N3	0.92774 (14)	0.33270 (12)	−0.15160 (13)	0.0482 (3)	
C1	0.41872 (16)	0.28397 (14)	0.27729 (14)	0.0405 (3)	
C2	0.25905 (18)	0.38446 (16)	0.36209 (16)	0.0513 (3)	
H2	0.1928	0.4583	0.3327	0.062*	
C3	0.2184 (2)	0.3528 (2)	0.49868 (16)	0.0626 (4)	
H3	0.1208	0.4020	0.5745	0.075*	
C4	0.3512 (2)	0.2339 (2)	0.50013 (16)	0.0604 (4)	
H4	0.3564	0.1912	0.5771	0.072*	
C5	0.47621 (18)	0.19029 (17)	0.36362 (15)	0.0478 (3)	
H5	0.5772	0.1144	0.3357	0.057*	
C6	0.3420 (3)	−0.0224 (3)	0.3165 (5)	0.0706 (9)	0.900 (10)
H6	0.4362	−0.1002	0.3064	0.085*	0.900 (10)
C7	0.3150 (4)	0.0551 (5)	0.2119 (2)	0.0650 (8)	0.900 (10)
H7	0.3887	0.0375	0.1200	0.078*	0.900 (10)
C8	0.1568 (4)	0.1645 (4)	0.2697 (4)	0.0647 (7)	0.900 (10)
H8	0.1083	0.2314	0.2229	0.078*	0.900 (10)
C9	0.0854 (3)	0.1543 (4)	0.4114 (4)	0.0695 (8)	0.900 (10)
H9	−0.0185	0.2131	0.4744	0.083*	0.900 (10)
C10	0.1991 (6)	0.0396 (4)	0.4404 (4)	0.0727 (9)	0.900 (10)
H10	0.1835	0.0094	0.5261	0.087*	0.900 (10)
C10A	0.261 (4)	−0.002 (3)	0.402 (3)	0.0727 (9)	0.100 (10)
H10A	0.2895	−0.0662	0.4602	0.087*	0.100 (10)
C6A	0.356 (2)	−0.012 (2)	0.259 (4)	0.0706 (9)	0.100 (10)
H6A	0.4573	−0.0852	0.2068	0.085*	0.100 (10)
C7A	0.270 (4)	0.107 (3)	0.2102 (18)	0.0650 (8)	0.100 (10)
H7A	0.3039	0.1269	0.1200	0.078*	0.100 (10)
C8A	0.121 (3)	0.192 (2)	0.323 (3)	0.0647 (7)	0.100 (10)
H8A	0.0413	0.2770	0.3197	0.078*	0.100 (10)
C9A	0.116 (3)	0.125 (4)	0.442 (2)	0.0695 (8)	0.100 (10)
H9A	0.0324	0.1577	0.5300	0.083*	0.100 (10)
C11	0.50334 (15)	0.28020 (13)	0.12904 (13)	0.0371 (3)	
C12	0.44094 (15)	0.35814 (13)	0.04066 (13)	0.0385 (3)	
H12	0.3359	0.4236	0.0637	0.046*	
C13	0.56798 (15)	0.31739 (13)	−0.08772 (13)	0.0372 (3)	
C14	0.57130 (16)	0.36385 (14)	−0.22026 (14)	0.0420 (3)	
C15	0.4087 (2)	0.5095 (2)	−0.32467 (17)	0.0594 (4)	
H15A	0.4244	0.4368	−0.3878	0.071*	
H15B	0.4878	0.5421	−0.3722	0.071*	
C16	0.2461 (3)	0.6295 (3)	−0.2784 (2)	0.0829 (6)	
H16A	0.2324	0.6692	−0.3573	0.124*	
H16B	0.2320	0.7008	−0.2162	0.124*	
H16C	0.1689	0.5959	−0.2317	0.124*	
C17	0.86328 (15)	0.14457 (15)	−0.17311 (15)	0.0467 (3)	
H17A	0.8703	0.1581	−0.2654	0.056*	
H17B	0.9026	0.0440	−0.1682	0.056*	
C18	0.96598 (15)	0.19558 (14)	−0.14829 (13)	0.0391 (3)	
C19	1.09484 (16)	0.10195 (16)	−0.12568 (15)	0.0478 (3)	
H19	1.1178	0.0071	−0.1236	0.057*	
C20	1.18884 (18)	0.15086 (19)	−0.10632 (17)	0.0576 (4)	
H20	1.2763	0.0896	−0.0912	0.069*	
C21	1.1511 (2)	0.2917 (2)	−0.10968 (17)	0.0596 (4)	
H21	1.2123	0.3281	−0.0974	0.072*	
C22	1.0197 (2)	0.37736 (17)	−0.13176 (17)	0.0564 (4)	
H22	0.9937	0.4729	−0.1329	0.068*	

### Atomic displacement parameters (Å^2^)

**Table:**

	*U*^11^	*U*^22^	*U*^33^	*U*^12^	*U*^13^	*U*^23^
Fe1	0.04003 (12)	0.05551 (14)	0.03486 (11)	−0.02580 (10)	−0.01357 (8)	0.00789 (8)
O1	0.0552 (6)	0.0692 (7)	0.0459 (6)	−0.0252 (6)	−0.0119 (5)	0.0163 (5)
O2	0.0487 (6)	0.0682 (7)	0.0461 (5)	−0.0272 (5)	−0.0272 (5)	0.0219 (5)
N1	0.0352 (5)	0.0441 (6)	0.0450 (6)	−0.0158 (5)	−0.0188 (5)	0.0100 (5)
N2	0.0313 (5)	0.0422 (6)	0.0426 (6)	−0.0150 (5)	−0.0143 (5)	0.0067 (4)
N3	0.0422 (6)	0.0419 (6)	0.0523 (7)	−0.0146 (5)	−0.0184 (5)	0.0070 (5)
C1	0.0396 (7)	0.0449 (7)	0.0419 (7)	−0.0223 (6)	−0.0188 (6)	0.0071 (5)
C2	0.0465 (8)	0.0486 (8)	0.0488 (8)	−0.0175 (6)	−0.0156 (6)	0.0004 (6)
C3	0.0619 (10)	0.0723 (11)	0.0408 (8)	−0.0296 (9)	−0.0101 (7)	−0.0079 (7)
C4	0.0748 (11)	0.0818 (11)	0.0391 (8)	−0.0436 (10)	−0.0296 (8)	0.0127 (7)
C5	0.0485 (8)	0.0587 (8)	0.0466 (7)	−0.0271 (7)	−0.0274 (6)	0.0122 (6)
C6	0.0692 (13)	0.0608 (11)	0.092 (3)	−0.0379 (10)	−0.0342 (17)	0.0061 (11)
C7	0.0596 (14)	0.084 (2)	0.0565 (10)	−0.0435 (15)	−0.0154 (10)	−0.0094 (12)
C8	0.0621 (16)	0.0932 (15)	0.0593 (18)	−0.0467 (13)	−0.0322 (16)	0.0100 (14)
C9	0.0490 (12)	0.0991 (17)	0.0668 (16)	−0.0475 (12)	−0.0158 (9)	0.0119 (13)
C10	0.078 (2)	0.084 (2)	0.0711 (15)	−0.057 (2)	−0.0255 (14)	0.0270 (13)
C10A	0.078 (2)	0.084 (2)	0.0711 (15)	−0.057 (2)	−0.0255 (14)	0.0270 (13)
C6A	0.0692 (13)	0.0608 (11)	0.092 (3)	−0.0379 (10)	−0.0342 (17)	0.0061 (11)
C7A	0.0596 (14)	0.084 (2)	0.0565 (10)	−0.0435 (15)	−0.0154 (10)	−0.0094 (12)
C8A	0.0621 (16)	0.0932 (15)	0.0593 (18)	−0.0467 (13)	−0.0322 (16)	0.0100 (14)
C9A	0.0490 (12)	0.0991 (17)	0.0668 (16)	−0.0475 (12)	−0.0158 (9)	0.0119 (13)
C11	0.0337 (6)	0.0380 (6)	0.0427 (7)	−0.0184 (5)	−0.0173 (5)	0.0082 (5)
C12	0.0312 (6)	0.0394 (6)	0.0441 (7)	−0.0156 (5)	−0.0167 (5)	0.0094 (5)
C13	0.0347 (6)	0.0370 (6)	0.0432 (7)	−0.0185 (5)	−0.0178 (5)	0.0094 (5)
C14	0.0450 (7)	0.0436 (7)	0.0443 (7)	−0.0255 (6)	−0.0202 (6)	0.0113 (5)
C15	0.0743 (11)	0.0762 (11)	0.0544 (9)	−0.0447 (9)	−0.0429 (8)	0.0302 (8)
C16	0.0765 (13)	0.1023 (16)	0.0912 (15)	−0.0423 (12)	−0.0582 (12)	0.0498 (13)
C17	0.0326 (6)	0.0469 (7)	0.0488 (8)	−0.0128 (6)	−0.0119 (6)	−0.0020 (6)
C18	0.0301 (6)	0.0416 (7)	0.0344 (6)	−0.0125 (5)	−0.0081 (5)	0.0045 (5)
C19	0.0378 (7)	0.0460 (7)	0.0515 (8)	−0.0150 (6)	−0.0170 (6)	0.0111 (6)
C20	0.0406 (8)	0.0699 (10)	0.0590 (9)	−0.0199 (7)	−0.0248 (7)	0.0108 (8)
C21	0.0504 (9)	0.0780 (11)	0.0536 (9)	−0.0368 (8)	−0.0158 (7)	−0.0007 (8)
C22	0.0579 (9)	0.0485 (8)	0.0572 (9)	−0.0270 (7)	−0.0167 (7)	0.0045 (7)

### Geometric parameters (Å, °)

**Table:**

Fe1—C8A	1.88 (2)	C8—C9	1.413 (3)
Fe1—C9A	1.94 (2)	C8—H8	0.9300
Fe1—C7A	1.979 (19)	C9—C10	1.403 (4)
Fe1—C7	2.029 (2)	C9—H9	0.9300
Fe1—C6	2.034 (2)	C10—H10	0.9300
Fe1—C4	2.0365 (16)	C10A—C9A	1.4200
Fe1—C5	2.0382 (14)	C10A—C6A	1.4200
Fe1—C3	2.0398 (16)	C10A—H10A	0.9300
Fe1—C1	2.0402 (13)	C6A—C7A	1.4200
Fe1—C2	2.0410 (16)	C6A—H6A	0.9300
Fe1—C10	2.044 (2)	C7A—C8A	1.4200
Fe1—C8	2.045 (2)	C7A—H7A	0.9300
O1—C14	1.2015 (17)	C8A—C9A	1.4200
O2—C14	1.3346 (17)	C8A—H8A	0.9300
O2—C15	1.4543 (17)	C9A—H9A	0.9300
N1—C11	1.3393 (16)	C11—C12	1.3973 (17)
N1—N2	1.3440 (16)	C12—C13	1.3754 (18)
N2—C13	1.3590 (16)	C12—H12	0.9300
N2—C17	1.4606 (17)	C13—C14	1.4710 (18)
N3—C22	1.328 (2)	C15—C16	1.487 (3)
N3—C18	1.3345 (18)	C15—H15A	0.9700
C1—C2	1.423 (2)	C15—H15B	0.9700
C1—C5	1.4268 (19)	C16—H16A	0.9600
C1—C11	1.4588 (18)	C16—H16B	0.9600
C2—C3	1.414 (2)	C16—H16C	0.9600
C2—H2	0.9300	C17—C18	1.5053 (19)
C3—C4	1.408 (3)	C17—H17A	0.9700
C3—H3	0.9300	C17—H17B	0.9700
C4—C5	1.423 (2)	C18—C19	1.3820 (19)
C4—H4	0.9300	C19—C20	1.377 (2)
C5—H5	0.9300	C19—H19	0.9300
C6—C7	1.403 (4)	C20—C21	1.374 (3)
C6—C10	1.420 (4)	C20—H20	0.9300
C6—H6	0.9300	C21—C22	1.377 (2)
C7—C8	1.413 (3)	C21—H21	0.9300
C7—H7	0.9300	C22—H22	0.9300
			
C8A—Fe1—C9A	43.7 (5)	C10—C6—Fe1	70.00 (13)
C8A—Fe1—C7A	43.1 (4)	C7—C6—H6	126.3
C9A—Fe1—C7A	71.8 (5)	C10—C6—H6	126.3
C8A—Fe1—C7	54.7 (8)	Fe1—C6—H6	125.7
C9A—Fe1—C7	72.9 (6)	C6—C7—C8	108.4 (2)
C7A—Fe1—C7	15.8 (9)	C6—C7—Fe1	69.98 (13)
C8A—Fe1—C6	75.5 (7)	C8—C7—Fe1	70.32 (12)
C9A—Fe1—C6	61.8 (9)	C6—C7—H7	125.8
C7A—Fe1—C6	54.6 (8)	C8—C7—H7	125.8
C7—Fe1—C6	40.41 (10)	Fe1—C7—H7	125.5
C8A—Fe1—C4	150.4 (9)	C9—C8—C7	107.8 (2)
C9A—Fe1—C4	117.5 (7)	C9—C8—Fe1	70.33 (12)
C7A—Fe1—C4	166.4 (9)	C7—C8—Fe1	69.10 (12)
C7—Fe1—C4	152.72 (14)	C9—C8—H8	126.1
C6—Fe1—C4	119.28 (13)	C7—C8—H8	126.1
C8A—Fe1—C5	165.0 (10)	Fe1—C8—H8	126.1
C9A—Fe1—C5	150.9 (10)	C10—C9—C8	107.9 (2)
C7A—Fe1—C5	126.5 (8)	C10—C9—Fe1	69.47 (12)
C7—Fe1—C5	118.16 (10)	C8—C9—Fe1	69.38 (11)
C6—Fe1—C5	107.71 (8)	C10—C9—H9	126.1
C4—Fe1—C5	40.88 (6)	C8—C9—H9	126.1
C8A—Fe1—C3	115.3 (7)	Fe1—C9—H9	126.7
C9A—Fe1—C3	107.9 (7)	C9—C10—C6	108.4 (2)
C7A—Fe1—C3	149.6 (10)	C9—C10—Fe1	70.51 (13)
C7—Fe1—C3	165.26 (15)	C6—C10—Fe1	69.25 (12)
C6—Fe1—C3	153.27 (15)	C9—C10—H10	125.8
C4—Fe1—C3	40.41 (7)	C6—C10—H10	125.8
C5—Fe1—C3	68.50 (7)	Fe1—C10—H10	126.0
C8A—Fe1—C1	125.2 (9)	C9A—C10A—C6A	108.0
C9A—Fe1—C1	166.9 (11)	C9A—C10A—Fe1	64.3 (8)
C7A—Fe1—C1	104.4 (6)	C6A—C10A—Fe1	71.0 (8)
C7—Fe1—C1	107.08 (8)	C9A—C10A—H10A	126.0
C6—Fe1—C1	126.89 (12)	C6A—C10A—H10A	126.0
C4—Fe1—C1	68.73 (6)	Fe1—C10A—H10A	130.3
C5—Fe1—C1	40.95 (5)	C7A—C6A—C10A	108.0
C3—Fe1—C1	68.62 (6)	C7A—C6A—Fe1	65.2 (8)
C8A—Fe1—C2	104.3 (7)	C10A—C6A—Fe1	69.2 (8)
C9A—Fe1—C2	128.5 (10)	C7A—C6A—H6A	126.0
C7A—Fe1—C2	114.9 (8)	C10A—C6A—H6A	126.0
C7—Fe1—C2	127.20 (13)	Fe1—C6A—H6A	131.2
C6—Fe1—C2	164.86 (15)	C6A—C7A—C8A	108.0
C4—Fe1—C2	68.20 (7)	C6A—C7A—Fe1	74.1 (8)
C5—Fe1—C2	68.57 (6)	C8A—C7A—Fe1	64.6 (8)
C3—Fe1—C2	40.54 (7)	C6A—C7A—H7A	126.0
C1—Fe1—C2	40.83 (6)	C8A—C7A—H7A	126.0
C8A—Fe1—C10	63.9 (8)	Fe1—C7A—H7A	126.7
C9A—Fe1—C10	26.9 (10)	C7A—C8A—C9A	108.0
C7A—Fe1—C10	74.5 (6)	C7A—C8A—Fe1	72.3 (8)
C7—Fe1—C10	67.96 (11)	C9A—C8A—Fe1	70.5 (9)
C6—Fe1—C10	40.75 (10)	C7A—C8A—H8A	126.0
C4—Fe1—C10	109.08 (9)	C9A—C8A—H8A	126.0
C5—Fe1—C10	128.22 (12)	Fe1—C8A—H8A	122.9
C3—Fe1—C10	119.57 (13)	C10A—C9A—C8A	108.0
C1—Fe1—C10	165.64 (15)	C10A—C9A—Fe1	74.5 (8)
C2—Fe1—C10	152.81 (15)	C8A—C9A—Fe1	65.8 (8)
C8A—Fe1—C8	15.3 (9)	C10A—C9A—H9A	126.0
C9A—Fe1—C8	53.2 (7)	C8A—C9A—H9A	126.0
C7A—Fe1—C8	28.0 (9)	Fe1—C9A—H9A	125.2
C7—Fe1—C8	40.58 (9)	N1—C11—C12	110.82 (11)
C6—Fe1—C8	68.13 (10)	N1—C11—C1	121.46 (11)
C4—Fe1—C8	165.62 (13)	C12—C11—C1	127.72 (12)
C5—Fe1—C8	152.11 (12)	C13—C12—C11	105.32 (11)
C3—Fe1—C8	127.79 (12)	C13—C12—H12	127.3
C1—Fe1—C8	118.02 (9)	C11—C12—H12	127.3
C2—Fe1—C8	107.86 (9)	N2—C13—C12	106.66 (11)
C10—Fe1—C8	67.67 (11)	N2—C13—C14	124.30 (12)
C14—O2—C15	115.57 (12)	C12—C13—C14	129.03 (12)
C11—N1—N2	105.42 (10)	O1—C14—O2	124.07 (13)
N1—N2—C13	111.78 (10)	O1—C14—C13	126.58 (13)
N1—N2—C17	118.09 (11)	O2—C14—C13	109.35 (11)
C13—N2—C17	130.10 (12)	O2—C15—C16	107.49 (15)
C22—N3—C18	116.95 (13)	O2—C15—H15A	110.2
C2—C1—C5	107.44 (13)	C16—C15—H15A	110.2
C2—C1—C11	124.84 (13)	O2—C15—H15B	110.2
C5—C1—C11	127.72 (13)	C16—C15—H15B	110.2
C2—C1—Fe1	69.61 (8)	H15A—C15—H15B	108.5
C5—C1—Fe1	69.45 (8)	C15—C16—H16A	109.5
C11—C1—Fe1	125.73 (9)	C15—C16—H16B	109.5
C3—C2—C1	108.29 (14)	H16A—C16—H16B	109.5
C3—C2—Fe1	69.68 (10)	C15—C16—H16C	109.5
C1—C2—Fe1	69.56 (8)	H16A—C16—H16C	109.5
C3—C2—H2	125.9	H16B—C16—H16C	109.5
C1—C2—H2	125.9	N2—C17—C18	111.90 (11)
Fe1—C2—H2	126.5	N2—C17—H17A	109.2
C4—C3—C2	108.23 (14)	C18—C17—H17A	109.2
C4—C3—Fe1	69.67 (9)	N2—C17—H17B	109.2
C2—C3—Fe1	69.77 (9)	C18—C17—H17B	109.2
C4—C3—H3	125.9	H17A—C17—H17B	107.9
C2—C3—H3	125.9	N3—C18—C19	122.68 (13)
Fe1—C3—H3	126.2	N3—C18—C17	116.92 (12)
C3—C4—C5	108.34 (14)	C19—C18—C17	120.39 (13)
C3—C4—Fe1	69.92 (9)	C20—C19—C18	119.17 (14)
C5—C4—Fe1	69.63 (8)	C20—C19—H19	120.4
C3—C4—H4	125.8	C18—C19—H19	120.4
C5—C4—H4	125.8	C21—C20—C19	118.81 (15)
Fe1—C4—H4	126.2	C21—C20—H20	120.6
C4—C5—C1	107.70 (14)	C19—C20—H20	120.6
C4—C5—Fe1	69.49 (9)	C20—C21—C22	117.97 (15)
C1—C5—Fe1	69.60 (8)	C20—C21—H21	121.0
C4—C5—H5	126.2	C22—C21—H21	121.0
C1—C5—H5	126.2	N3—C22—C21	124.42 (15)
Fe1—C5—H5	126.3	N3—C22—H22	117.8
C7—C6—C10	107.5 (2)	C21—C22—H22	117.8
C7—C6—Fe1	69.61 (13)		
			
C11—N1—N2—C13	0.02 (14)	C7—Fe1—C9—C10	81.54 (18)
C11—N1—N2—C17	178.37 (11)	C6—Fe1—C9—C10	37.80 (15)
C8A—Fe1—C1—C2	69.3 (10)	C4—Fe1—C9—C10	−72.9 (2)
C9A—Fe1—C1—C2	40 (3)	C5—Fe1—C9—C10	−37.1 (5)
C7A—Fe1—C1—C2	111.5 (10)	C3—Fe1—C9—C10	−113.8 (2)
C7—Fe1—C1—C2	127.72 (16)	C1—Fe1—C9—C10	166.4 (2)
C6—Fe1—C1—C2	167.79 (16)	C2—Fe1—C9—C10	−157.0 (2)
C4—Fe1—C1—C2	−80.86 (10)	C8—Fe1—C9—C10	119.4 (2)
C5—Fe1—C1—C2	−118.74 (12)	C8A—Fe1—C9—C8	18.3 (14)
C3—Fe1—C1—C2	−37.33 (9)	C9A—Fe1—C9—C8	−144 (3)
C10—Fe1—C1—C2	−164.8 (3)	C7A—Fe1—C9—C8	−21.3 (10)
C8—Fe1—C1—C2	85.16 (15)	C7—Fe1—C9—C8	−37.88 (15)
C8A—Fe1—C1—C5	−172.0 (10)	C6—Fe1—C9—C8	−81.63 (17)
C9A—Fe1—C1—C5	159 (3)	C4—Fe1—C9—C8	167.67 (17)
C7A—Fe1—C1—C5	−129.8 (10)	C5—Fe1—C9—C8	−156.5 (3)
C7—Fe1—C1—C5	−113.53 (16)	C3—Fe1—C9—C8	126.75 (19)
C6—Fe1—C1—C5	−73.47 (18)	C1—Fe1—C9—C8	47.0 (3)
C4—Fe1—C1—C5	37.88 (9)	C2—Fe1—C9—C8	83.53 (19)
C3—Fe1—C1—C5	81.41 (10)	C10—Fe1—C9—C8	−119.4 (2)
C2—Fe1—C1—C5	118.74 (12)	C8—C9—C10—C6	−0.2 (2)
C10—Fe1—C1—C5	−46.0 (4)	Fe1—C9—C10—C6	−59.10 (15)
C8—Fe1—C1—C5	−156.10 (14)	C8—C9—C10—Fe1	58.94 (15)
C8A—Fe1—C1—C11	−49.6 (10)	C7—C6—C10—C9	0.1 (2)
C9A—Fe1—C1—C11	−79 (3)	Fe1—C6—C10—C9	59.88 (16)
C7A—Fe1—C1—C11	−7.4 (10)	C7—C6—C10—Fe1	−59.77 (15)
C7—Fe1—C1—C11	8.82 (19)	C8A—Fe1—C10—C9	−21.3 (9)
C6—Fe1—C1—C11	48.9 (2)	C9A—Fe1—C10—C9	14.1 (13)
C4—Fe1—C1—C11	160.23 (14)	C7A—Fe1—C10—C9	−66.2 (10)
C5—Fe1—C1—C11	122.35 (15)	C7—Fe1—C10—C9	−81.50 (16)
C3—Fe1—C1—C11	−156.24 (14)	C6—Fe1—C10—C9	−119.4 (2)
C2—Fe1—C1—C11	−118.91 (15)	C4—Fe1—C10—C9	127.5 (2)
C10—Fe1—C1—C11	76.3 (4)	C5—Fe1—C10—C9	169.27 (16)
C8—Fe1—C1—C11	−33.75 (18)	C3—Fe1—C10—C9	84.4 (2)
C5—C1—C2—C3	−0.28 (17)	C1—Fe1—C10—C9	−153.8 (3)
C11—C1—C2—C3	179.13 (13)	C2—Fe1—C10—C9	48.3 (3)
Fe1—C1—C2—C3	59.10 (11)	C8—Fe1—C10—C9	−37.51 (15)
C5—C1—C2—Fe1	−59.38 (10)	C8A—Fe1—C10—C6	98.2 (9)
C11—C1—C2—Fe1	120.03 (13)	C9A—Fe1—C10—C6	133.5 (14)
C8A—Fe1—C2—C3	112.4 (10)	C7A—Fe1—C10—C6	53.2 (10)
C9A—Fe1—C2—C3	71.0 (9)	C7—Fe1—C10—C6	37.94 (15)
C7A—Fe1—C2—C3	157.0 (10)	C4—Fe1—C10—C6	−113.0 (2)
C7—Fe1—C2—C3	168.65 (15)	C5—Fe1—C10—C6	−71.3 (2)
C6—Fe1—C2—C3	−160.0 (3)	C3—Fe1—C10—C6	−156.15 (18)
C4—Fe1—C2—C3	−37.43 (10)	C1—Fe1—C10—C6	−34.4 (4)
C5—Fe1—C2—C3	−81.56 (11)	C2—Fe1—C10—C6	167.7 (2)
C1—Fe1—C2—C3	−119.68 (13)	C8—Fe1—C10—C6	81.93 (17)
C10—Fe1—C2—C3	52.1 (2)	C8A—Fe1—C10A—C9A	−40.0 (5)
C8—Fe1—C2—C3	127.86 (15)	C7A—Fe1—C10A—C9A	−86.0 (6)
C8A—Fe1—C2—C1	−127.9 (10)	C7—Fe1—C10A—C9A	−99.6 (9)
C9A—Fe1—C2—C1	−169.3 (9)	C6—Fe1—C10A—C9A	−136.3 (16)
C7A—Fe1—C2—C1	−83.3 (10)	C4—Fe1—C10A—C9A	108.6 (10)
C7—Fe1—C2—C1	−71.67 (15)	C5—Fe1—C10A—C9A	152.9 (10)
C6—Fe1—C2—C1	−40.4 (4)	C3—Fe1—C10A—C9A	67.4 (11)
C4—Fe1—C2—C1	82.25 (9)	C1—Fe1—C10A—C9A	−168.5 (12)
C5—Fe1—C2—C1	38.13 (8)	C2—Fe1—C10A—C9A	27 (3)
C3—Fe1—C2—C1	119.68 (13)	C10—Fe1—C10A—C9A	21 (2)
C10—Fe1—C2—C1	171.79 (19)	C8—Fe1—C10A—C9A	−56.2 (9)
C8—Fe1—C2—C1	−112.46 (14)	C8A—Fe1—C10A—C6A	82.0 (6)
C1—C2—C3—C4	0.23 (19)	C9A—Fe1—C10A—C6A	121.9 (5)
Fe1—C2—C3—C4	59.26 (12)	C7A—Fe1—C10A—C6A	36.0 (4)
C1—C2—C3—Fe1	−59.03 (10)	C7—Fe1—C10A—C6A	22.4 (9)
C8A—Fe1—C3—C4	158.1 (11)	C6—Fe1—C10A—C6A	−14.4 (15)
C9A—Fe1—C3—C4	111.6 (11)	C4—Fe1—C10A—C6A	−129.5 (10)
C7A—Fe1—C3—C4	−164.0 (13)	C5—Fe1—C10A—C6A	−85.1 (9)
C7—Fe1—C3—C4	−157.5 (3)	C3—Fe1—C10A—C6A	−170.6 (10)
C6—Fe1—C3—C4	49.1 (2)	C1—Fe1—C10A—C6A	−46.6 (12)
C5—Fe1—C3—C4	−37.73 (10)	C2—Fe1—C10A—C6A	149 (3)
C1—Fe1—C3—C4	−81.88 (10)	C10—Fe1—C10A—C6A	143 (2)
C2—Fe1—C3—C4	−119.47 (14)	C8—Fe1—C10A—C6A	65.8 (9)
C10—Fe1—C3—C4	85.02 (17)	C9A—C10A—C6A—C7A	0.0
C8—Fe1—C3—C4	168.55 (14)	Fe1—C10A—C6A—C7A	−53.5 (8)
C8A—Fe1—C3—C2	−82.4 (11)	C9A—C10A—C6A—Fe1	53.5 (8)
C9A—Fe1—C3—C2	−128.9 (11)	C8A—Fe1—C6A—C7A	39.2 (4)
C7A—Fe1—C3—C2	−44.5 (13)	C9A—Fe1—C6A—C7A	85.9 (7)
C7—Fe1—C3—C2	−38.0 (4)	C7—Fe1—C6A—C7A	−7.8 (15)
C6—Fe1—C3—C2	168.57 (18)	C6—Fe1—C6A—C7A	145 (2)
C4—Fe1—C3—C2	119.47 (14)	C4—Fe1—C6A—C7A	−165.1 (10)
C5—Fe1—C3—C2	81.74 (10)	C5—Fe1—C6A—C7A	−124.9 (10)
C1—Fe1—C3—C2	37.59 (9)	C3—Fe1—C6A—C7A	163 (3)
C10—Fe1—C3—C2	−155.51 (15)	C1—Fe1—C6A—C7A	−81.2 (10)
C8—Fe1—C3—C2	−71.98 (15)	C2—Fe1—C6A—C7A	−45.9 (13)
C2—C3—C4—C5	−0.10 (19)	C10—Fe1—C6A—C7A	110.7 (10)
Fe1—C3—C4—C5	59.23 (11)	C8—Fe1—C6A—C7A	27.2 (9)
C2—C3—C4—Fe1	−59.32 (12)	C8A—Fe1—C6A—C10A	−83.4 (7)
C8A—Fe1—C4—C3	−43.0 (16)	C9A—Fe1—C6A—C10A	−36.8 (4)
C9A—Fe1—C4—C3	−85.7 (12)	C7A—Fe1—C6A—C10A	−122.6 (5)
C7A—Fe1—C4—C3	143 (3)	C7—Fe1—C6A—C10A	−130.4 (14)
C7—Fe1—C4—C3	167.7 (2)	C6—Fe1—C6A—C10A	23 (2)
C6—Fe1—C4—C3	−157.06 (16)	C4—Fe1—C6A—C10A	72.2 (9)
C5—Fe1—C4—C3	119.54 (14)	C5—Fe1—C6A—C10A	112.4 (10)
C1—Fe1—C4—C3	81.59 (10)	C3—Fe1—C6A—C10A	40 (3)
C2—Fe1—C4—C3	37.55 (10)	C1—Fe1—C6A—C10A	156.1 (10)
C10—Fe1—C4—C3	−113.54 (18)	C2—Fe1—C6A—C10A	−168.6 (13)
C8—Fe1—C4—C3	−39.2 (4)	C10—Fe1—C6A—C10A	−11.9 (9)
C8A—Fe1—C4—C5	−162.6 (16)	C8—Fe1—C6A—C10A	−95.4 (9)
C9A—Fe1—C4—C5	154.8 (12)	C10A—C6A—C7A—C8A	0.0
C7A—Fe1—C4—C5	24 (3)	Fe1—C6A—C7A—C8A	−55.9 (8)
C7—Fe1—C4—C5	48.2 (2)	C10A—C6A—C7A—Fe1	55.9 (8)
C6—Fe1—C4—C5	83.40 (16)	C8A—Fe1—C7A—C6A	−119.4 (5)
C3—Fe1—C4—C5	−119.54 (14)	C9A—Fe1—C7A—C6A	−79.1 (6)
C1—Fe1—C4—C5	−37.95 (9)	C7—Fe1—C7A—C6A	12 (2)
C2—Fe1—C4—C5	−81.99 (10)	C6—Fe1—C7A—C6A	−11.3 (10)
C10—Fe1—C4—C5	126.92 (17)	C4—Fe1—C7A—C6A	56 (3)
C8—Fe1—C4—C5	−158.7 (3)	C5—Fe1—C7A—C6A	75.2 (10)
C3—C4—C5—C1	−0.07 (18)	C3—Fe1—C7A—C6A	−173.7 (13)
Fe1—C4—C5—C1	59.34 (10)	C1—Fe1—C7A—C6A	114.0 (11)
C3—C4—C5—Fe1	−59.41 (12)	C2—Fe1—C7A—C6A	156.1 (10)
C2—C1—C5—C4	0.21 (16)	C10—Fe1—C7A—C6A	−51.2 (10)
C11—C1—C5—C4	−179.17 (13)	C8—Fe1—C7A—C6A	−122.7 (13)
Fe1—C1—C5—C4	−59.27 (11)	C9A—Fe1—C7A—C8A	40.3 (4)
C2—C1—C5—Fe1	59.48 (10)	C7—Fe1—C7A—C8A	132 (2)
C11—C1—C5—Fe1	−119.90 (13)	C6—Fe1—C7A—C8A	108.1 (11)
C8A—Fe1—C5—C4	145 (3)	C4—Fe1—C7A—C8A	175 (3)
C9A—Fe1—C5—C4	−51.1 (16)	C5—Fe1—C7A—C8A	−165.4 (11)
C7A—Fe1—C5—C4	−173.2 (11)	C3—Fe1—C7A—C8A	−54.4 (13)
C7—Fe1—C5—C4	−157.20 (17)	C1—Fe1—C7A—C8A	−126.6 (12)
C6—Fe1—C5—C4	−114.56 (18)	C2—Fe1—C7A—C8A	−84.5 (11)
C3—Fe1—C5—C4	37.31 (10)	C10—Fe1—C7A—C8A	68.2 (11)
C1—Fe1—C5—C4	119.04 (13)	C8—Fe1—C7A—C8A	−3.3 (15)
C2—Fe1—C5—C4	81.03 (11)	C6A—C7A—C8A—C9A	0.0
C10—Fe1—C5—C4	−74.09 (19)	Fe1—C7A—C8A—C9A	−61.8 (8)
C8—Fe1—C5—C4	168.9 (2)	C6A—C7A—C8A—Fe1	61.8 (8)
C8A—Fe1—C5—C1	26 (3)	C9A—Fe1—C8A—C7A	−117.2 (4)
C9A—Fe1—C5—C1	−170.2 (16)	C7—Fe1—C8A—C7A	−14.5 (9)
C7A—Fe1—C5—C1	67.8 (11)	C6—Fe1—C8A—C7A	−53.1 (10)
C7—Fe1—C5—C1	83.77 (16)	C4—Fe1—C8A—C7A	−177.8 (13)
C6—Fe1—C5—C1	126.40 (17)	C5—Fe1—C8A—C7A	51 (2)
C4—Fe1—C5—C1	−119.04 (13)	C3—Fe1—C8A—C7A	152.9 (10)
C3—Fe1—C5—C1	−81.73 (10)	C1—Fe1—C8A—C7A	72.1 (10)
C2—Fe1—C5—C1	−38.01 (8)	C2—Fe1—C8A—C7A	111.2 (11)
C10—Fe1—C5—C1	166.87 (16)	C10—Fe1—C8A—C7A	−95.0 (10)
C8—Fe1—C5—C1	49.9 (2)	C8—Fe1—C8A—C7A	6(3)
C8A—Fe1—C6—C7	51.8 (10)	C7A—Fe1—C8A—C9A	117.2 (4)
C9A—Fe1—C6—C7	96.6 (9)	C7—Fe1—C8A—C9A	102.7 (10)
C7A—Fe1—C6—C7	9.7 (9)	C6—Fe1—C8A—C9A	64.1 (11)
C4—Fe1—C6—C7	−155.95 (15)	C4—Fe1—C8A—C9A	−60.6 (13)
C5—Fe1—C6—C7	−112.91 (16)	C5—Fe1—C8A—C9A	169 (2)
C3—Fe1—C6—C7	169.88 (18)	C3—Fe1—C8A—C9A	−89.9 (11)
C1—Fe1—C6—C7	−71.64 (17)	C1—Fe1—C8A—C9A	−170.7 (11)
C2—Fe1—C6—C7	−39.7 (4)	C2—Fe1—C8A—C9A	−131.5 (12)
C10—Fe1—C6—C7	118.5 (2)	C10—Fe1—C8A—C9A	22.2 (10)
C8—Fe1—C6—C7	37.73 (13)	C8—Fe1—C8A—C9A	123 (3)
C8A—Fe1—C6—C10	−66.6 (10)	C6A—C10A—C9A—C8A	0.0
C9A—Fe1—C6—C10	−21.8 (9)	Fe1—C10A—C9A—C8A	57.5 (8)
C7A—Fe1—C6—C10	−108.8 (10)	C6A—C10A—C9A—Fe1	−57.5 (8)
C7—Fe1—C6—C10	−118.5 (2)	C7A—C8A—C9A—C10A	0.0
C4—Fe1—C6—C10	85.60 (19)	Fe1—C8A—C9A—C10A	−63.0 (8)
C5—Fe1—C6—C10	128.64 (19)	C7A—C8A—C9A—Fe1	63.0 (8)
C3—Fe1—C6—C10	51.4 (3)	C8A—Fe1—C9A—C10A	118.4 (4)
C1—Fe1—C6—C10	169.91 (16)	C7A—Fe1—C9A—C10A	78.7 (6)
C2—Fe1—C6—C10	−158.1 (3)	C7—Fe1—C9A—C10A	62.1 (10)
C8—Fe1—C6—C10	−80.72 (17)	C6—Fe1—C9A—C10A	19.7 (10)
C10—C6—C7—C8	0.0 (2)	C4—Fe1—C9A—C10A	−90.6 (10)
Fe1—C6—C7—C8	−60.04 (14)	C5—Fe1—C9A—C10A	−55.5 (13)
C10—C6—C7—Fe1	60.02 (15)	C3—Fe1—C9A—C10A	−133.4 (11)
C8A—Fe1—C7—C6	−111.1 (11)	C1—Fe1—C9A—C10A	154 (3)
C9A—Fe1—C7—C6	−66.3 (11)	C2—Fe1—C9A—C10A	−173.6 (11)
C7A—Fe1—C7—C6	−150 (2)	C10—Fe1—C9A—C10A	−12.8 (15)
C4—Fe1—C7—C6	50.9 (2)	C8—Fe1—C9A—C10A	102.4 (10)
C5—Fe1—C7—C6	84.46 (16)	C7A—Fe1—C9A—C8A	−39.8 (4)
C3—Fe1—C7—C6	−161.9 (3)	C7—Fe1—C9A—C8A	−56.4 (10)
C1—Fe1—C7—C6	127.43 (16)	C6—Fe1—C9A—C8A	−98.7 (10)
C2—Fe1—C7—C6	167.91 (15)	C4—Fe1—C9A—C8A	151.0 (10)
C10—Fe1—C7—C6	−38.25 (14)	C5—Fe1—C9A—C8A	−173.9 (13)
C8—Fe1—C7—C6	−119.19 (19)	C3—Fe1—C9A—C8A	108.2 (11)
C8A—Fe1—C7—C8	8.1 (11)	C1—Fe1—C9A—C8A	36 (3)
C9A—Fe1—C7—C8	52.9 (11)	C2—Fe1—C9A—C8A	68.0 (11)
C7A—Fe1—C7—C8	−31 (2)	C10—Fe1—C9A—C8A	−131.2 (15)
C6—Fe1—C7—C8	119.19 (19)	C8—Fe1—C9A—C8A	−16.0 (9)
C4—Fe1—C7—C8	170.05 (17)	N2—N1—C11—C12	0.02 (14)
C5—Fe1—C7—C8	−156.35 (15)	N2—N1—C11—C1	−179.75 (11)
C3—Fe1—C7—C8	−42.7 (4)	C2—C1—C11—N1	170.29 (13)
C1—Fe1—C7—C8	−113.38 (17)	C5—C1—C11—N1	−10.4 (2)
C2—Fe1—C7—C8	−72.89 (18)	Fe1—C1—C11—N1	−100.98 (14)
C10—Fe1—C7—C8	80.94 (17)	C2—C1—C11—C12	−9.4 (2)
C6—C7—C8—C9	−0.1 (2)	C5—C1—C11—C12	169.84 (13)
Fe1—C7—C8—C9	−59.92 (14)	Fe1—C1—C11—C12	79.29 (17)
C6—C7—C8—Fe1	59.84 (14)	N1—C11—C12—C13	−0.06 (15)
C8A—Fe1—C8—C9	−35 (3)	C1—C11—C12—C13	179.69 (12)
C9A—Fe1—C8—C9	11.0 (12)	N1—N2—C13—C12	−0.06 (14)
C7A—Fe1—C8—C9	136.2 (15)	C17—N2—C13—C12	−178.15 (13)
C7—Fe1—C8—C9	119.0 (2)	N1—N2—C13—C14	−179.11 (11)
C6—Fe1—C8—C9	81.40 (17)	C17—N2—C13—C14	2.8 (2)
C4—Fe1—C8—C9	−42.4 (4)	C11—C12—C13—N2	0.07 (14)
C5—Fe1—C8—C9	168.09 (18)	C11—C12—C13—C14	179.06 (12)
C3—Fe1—C8—C9	−73.6 (2)	C15—O2—C14—O1	−1.6 (2)
C1—Fe1—C8—C9	−157.33 (17)	C15—O2—C14—C13	179.15 (12)
C2—Fe1—C8—C9	−114.14 (19)	N2—C13—C14—O1	−3.4 (2)
C10—Fe1—C8—C9	37.26 (16)	C12—C13—C14—O1	177.78 (14)
C8A—Fe1—C8—C7	−154 (3)	N2—C13—C14—O2	175.85 (12)
C9A—Fe1—C8—C7	−107.9 (12)	C12—C13—C14—O2	−2.98 (19)
C7A—Fe1—C8—C7	17.3 (15)	C14—O2—C15—C16	−166.88 (14)
C6—Fe1—C8—C7	−37.58 (14)	N1—N2—C17—C18	−69.18 (16)
C4—Fe1—C8—C7	−161.4 (3)	C13—N2—C17—C18	108.81 (16)
C5—Fe1—C8—C7	49.1 (3)	C22—N3—C18—C19	−0.1 (2)
C3—Fe1—C8—C7	167.39 (17)	C22—N3—C18—C17	−179.02 (13)
C1—Fe1—C8—C7	83.70 (18)	N2—C17—C18—N3	−58.92 (17)
C2—Fe1—C8—C7	126.89 (18)	N2—C17—C18—C19	122.13 (14)
C10—Fe1—C8—C7	−81.72 (17)	N3—C18—C19—C20	−0.3 (2)
C7—C8—C9—C10	0.1 (2)	C17—C18—C19—C20	178.57 (13)
Fe1—C8—C9—C10	−59.00 (16)	C18—C19—C20—C21	0.2 (2)
C7—C8—C9—Fe1	59.14 (14)	C19—C20—C21—C22	0.3 (2)
C8A—Fe1—C9—C10	137.7 (15)	C18—N3—C22—C21	0.7 (2)
C9A—Fe1—C9—C10	−25 (3)	C20—C21—C22—N3	−0.8 (3)
C7A—Fe1—C9—C10	98.1 (10)		
